# Energy and Micronutrient Intake One Year After Sleeve Gastrectomy Versus Roux-en-Y Gastric Bypass: A Substudy of the Bypass Equipoise Sleeve Trial

**DOI:** 10.1007/s11695-026-08550-3

**Published:** 2026-03-06

**Authors:** Emma Lundgren, Anna Laurenius, Suzanne Hedberg, Anders Thorell, Moa Hägg, Susanne Grehn, Kristina Spetz, Torsten Olbers, Ellen Andersson

**Affiliations:** 1 Department of Surgery, Vrinnevi Hospital, Norrköping, Sweden; 2 Department of Biomedical and Clinical Sciences, Linköping University, Linköping, Sweden; 3 Department of Surgery, Institute of Clinical Science, Sahlgrenska Academy, University of Gothenburg, Gothenburg, Sweden; 4 Department of Gastroenterology and Hepatology, Sahlgrenska University Hospital, Gothenburg, Region Västra Götaland, Sweden; 5 Department of Surgery, Department of Clinical Science, Sahlgrenska Academy, University of Gothenburg, Gothenburg, Sweden; 6 Department of Surgery (Östra Sjukhuset), Sahlgrenska University Hospital, Gothenburg, Sweden; 7 Department of Surgery & Anaesthesiology, Ersta Hospital, Stockholm, Sweden; 8 Department of Clinical Sciences, Danderyd Hospital, Karolinska Institutet, Stockholm, Sweden

## Abstract

**Introduction/Purpose:**

Sleeve Gastrectomy (SG) and Roux-en-Y Gastric Bypass (RYGB) entail risks of vitamin and mineral deficiencies, and guidelines recommend lifelong supplementation. It remains unclear if postoperative intake of micronutrients differs between surgical methods. This exploratory cross-sectional substudy aimed to evaluate whether the reported intake of vitamins and minerals in diet and supplements differs between SG and RYGB one year after surgery.

**Methods:**

Between April 2017 and May 2023, 285 participants from the randomized controlled multicenter trial “*Bypass Equipoise Sleeve Trial*” (BEST) were consecutively included at seven metabolic bariatric surgery centers in Sweden. The intake of vitamins and minerals from diet and supplements was self-reported at one-year follow-up and calculated in Dietist Net^®^. The Goldberg cut-off method was used to exclude under-reporters.

**Results:**

One year after surgery, the reported daily energy intake was 1673 kcal and 1651 kcal, after SG and RYGB, respectively. Compared with reference data on Swedish dietary habits from 2010 to 2011, participants reported daily intakes from diet below the daily recommended intake (RI) for a greater number of micronutrients. The reported intake of vitamin C from diet was 53 mg (SG) versus 72 mg (RYGB) (*p* = 0.001). Following both procedures most participants reported supplement intake consistent with the recommendations. The reported intake from supplements was 580 Retinol equivalents (RE) (SG) versus 656 RE (RYGB) for vitamin A (*p* = 0.017), and 374 µg (SG) versus 436 µg for folic acid (*p* = 0.014).

**Conclusion:**

Overall, the reported intake of energy and vitamins and minerals from diet and supplements did not differ between SG and RYGB one year after bariatric surgery. Minor differences were observed, with a higher intake of vitamin C from diet, and vitamin A and folic acid from supplements after RYGB.

**Supplementary Information:**

The online version contains supplementary material available at 10.1007/s11695-026-08550-3.

## Key points

Reported intake of vitamins and minerals from diet and supplements was generally similar and satisfactory one year after SG and RYGB.

Reported energy intake was comparable between SG and RYGB one year after bariatric surgery.

Most participants reported consistent use of vitamin and mineral supplements one year after bariatric surgery.

**Supplementary Information:**

The online version contains supplementary material available at 10.1007/s11695-026-08550-3.

## Introduction

Globally, a large and increasing number of individuals have undergone metabolic and bariatric surgery (MBS). The health-benefits of MBS are well-documented [1–3], and it has for a long time been the only effective treatment option of the chronic disease obesity. However, when choosing surgical procedure, the long-term adverse effects must be taken into consideration.

Laparoscopic sleeve gastrectomy (SG) and Roux-en-Y gastric bypass (RYGB) are the two most common bariatric procedures [4], both associated with a low risk of perioperative complications and very low perioperative mortality [4, 5]. In addition, in the short term both methods demonstrate similar efficacy in terms of weight loss and their impact on comorbidities [6, 7].

The anatomy of the gastrointestinal tract is altered in MBS, leading to a more rapid exposure of nutrients to the small intestine, which in turn induces changes in the profiles of hunger and satiety hormones. This will reduce calorie intake and thus induce long-term weight loss [8]. However, reduced gastrointestinal absorption of vitamins and minerals, and changes in dietary intake [9, 10] pose the risk of developing micronutrient deficiencies [11] that may result in complications, such as neuropathy, anemia, and osteoporosis [12, 13]. To prevent deficiencies and complications after MBS, lifelong vitamin and mineral supplementation, along with a diet rich in micronutrients is recommended [14, 15].

The comparative nutrient intake and food choices after SG and RYGB remain insufficiently defined [16–20]. SG has been suggested to result in fewer deficiencies than RYGB, presumably due to the fact that there is no bypass of any part of the intestine in SG, and SG seems to result in a lower postoperative increase in levels of osteocalcin and parathyroid hormone (PTH) compared to RYGB [21] as well as a lower prevalence of anemia [22]. However, micronutrient absorption may still be affected due to reduced secretion of gastric acid and intrinsic factor [13, 17, 23, 24].

Randomized trials comparing SG and RYBG have reported a lower intake of micronutrients from diet after SG compared to RYGB one year after surgery [25], but no significant differences in micronutrient deficiencies at three [26] and five years after surgery [27]. Given the uncertainty regarding differences in micronutrient intake and development of deficiencies after SG and RYGB, the clinical recommendations for micronutrient supplementation have been largely similar [15, 28–31].

The aim of this exploratory cross-sectional study was to compare the reported intake of vitamins and minerals from diet and supplements one year after surgery, in a randomized trial of SG and RYGB.

## Materials and Methods

### Study Design and Recruitment

Between April 2017 and May 2023, 300 participants in the randomized multicenter trial *Bypass Equipoise Sleeve Trial* (BEST) [32], were consecutively included in this substudy on nutritional intake at seven MBS centre’s in Sweden; Ersta hospital, Stockholm, Falun/Mora, Göteborg, Kalmar, Lindesberg/Örebro and Norrköping. The design of the BEST trial has been described in detail elsewhere [32]. Briefly, the BEST trial is a randomized controlled study comparing SG and RYGB, in which adult patients in Sweden and Norway with a BMI of 35–50 kg/m² were randomized in a 1:1 ratio to undergo either RYGB or SG [33].

Inclusion and data collection in the current study was conducted in conjunction to a routine out-patient visit one year after the surgery. All patients eligible for participation in the study received written information about the study by mail three weeks prior to routine follow-up. Three to four working days later, the patients were contacted by telephone and if they agreed to participate, a consent form was signed and returned. The participants then received a food diary with complementary questionnaires, to be completed before the routine clinical visit.

Anthropometric data were retrieved from the Scandinavian Obesity Surgery Register (SOReg) a quality registry for bariatric surgery in Sweden. SOReg covers more than 98% of all bariatric surgical procedures performed in Sweden and the registry has a high validity of registered data [34, 35].

### Vitamin and Mineral Supplementation

Swedish healthcare is publicly funded and includes a pharmaceutical benefit that subsidizes prescription medicines. All patients received the same prescription and recommendation of micronutrient supplementation at discharge after surgery, regardless of procedure, in accordance with the Nordic Guidelines [14] including a daily 1 mg of vitamin B12, 50 mg of iron and combined calcium-vitamin D (minimum 500 mg/20 µg). Additionally, patients were recommended an over-the-counter (OTC) multivitamin/mineral tablet. As an alternative, patients could choose an OTC specially designed preparation containing all required supplements in a single formulation. The latter is not included in the subsidized pharmaceutical benefit.

Self-reported intake of vitamin and mineral supplements, including frequency and dosage, was used to assess daily intake. Incomplete questionnaires were treated as missing data for both supplementation and total intake of vitamins and minerals.

### Dietary Assessment

Dietary micronutrient intake was assessed by a 4-day food record (three weekdays, and one of either Saturday or Sunday). The participants were asked to weigh or measure all food and beverages consumed. To reduce the risk of misreporting, the food diary was recorded with a dietitian trained in dietary assessment during the follow-up appointment one year after surgery. The average daily intake of energy and each micronutrient was assessed using Dietist Net^®^ version 20,160,217–20,230,613, a computerized dietary analysis software based on the Swedish Food Agency’s database, commonly used in clinical nutrition practice in Sweden [36].

As a comparison group the reported intake from Swedish adults aged 18–64 years in a national survey (Swedish dietary habits) from 2010 to 2011 was used. This survey involved 1,797 participants in four age groups (18–30, 31–44, 45–64, 65–80 years) who recorded their food intake for 4 days [37]. The sample was stratified by gender, age group, and region. The selection was evenly distributed across 12 months to account for seasonal variation in food choices. The age group 65–80 years was excluded (*n* = 367) to align the average age between the intervention and comparison groups.

### Classification of Under Reporters

The Goldberg cut-off method, described in detail elsewhere [38–40], was used to detect under- and over-reporters of energy intake. The method compares the reported energy intake with energy expenditure, based on the principle that energy intake should equal energy expenditure indicating when the reported intake is too low or too high to accurately reflect habitual consumption, after accounting for variations in both energy expenditure and energy intake. Reported intake within the upper and lower thresholds is classified as acceptable reports [38–40]. Basal metabolic rate (BMR) was calculated using the Mifflin-St Jeor equation [41], which takes age, gender, weight, and height into account. Since physical activity was not recorded, a physical activity level (PAL) of 1.4 (very light) was chosen based on suggestions from previous reports and to account for the tendency of the Mifflin–St Jeor equations to overestimate BMR in this population [41–44].

### Statistical Analysis

Data are presented as means ± SD or frequency (%), if not otherwise stated. Data in relation to the comparison group (Swedish dietary habits) are illustrated descriptively. The differences in intake between the SG and RYGB groups were calculated with unpaired t-test. All statistical tests were 2-sided, and *P* < 0.05 was considered statistically significant. SPSS (IBM SPSS Statistics for Windows, Version 29.0.2.0 Armonk, NY: IBM Corp) was used for statistical calculations.

### Ethical Approval

This trial was conducted in accordance with the ethical standards of the Declaration of Helsinki [45] and approved by the Regional Ethical Review Board in Gothenburg, Sweden (number 219 − 17 and amendment number 291 − 18).

## Results

### Demographics and Characteristics

A total of 285 individuals out of 693 eligible (41%) agreed to participate in the study (*n* = 148 SG and *n* = 137 RYGB). An analysis for patients eligible but not included was conducted at one surgical center. Of the 197 patients eligible at this center, 109 (55%) were included in the study. The reasons for non-inclusion included language barriers (*n* = 2), time constraints (*n* = 10), missed appointments or failure to be approached (*n* = 10), without providing a reason (*n* = 44), and missing documentation about being approached (*n* = 22).

Baseline characteristics are given in Table 1. There were no clinically relevant differences between SG and RYGB groups as assessed at baseline. One year after surgery, participants who underwent SG had a significantly less total weight loss and reduction in BMI compared to participants who underwent RYGB.

**Table 1:** Demographics at baseline and 1 year after randomization to Sleeve Gastrectomy and Roux-en-Y Gastric Bypass

Demographics	Sleeve Gastrectomy (*n* = 148)	Roux-en-Y Gastric Bypass (*n* = 137)	*p*-values
Female	114 (77.0%)	105 (76.6%)	
Male	34 (23.0%)	32 (23.4%)	
Baseline			
Bodyweight, kg	116.2 ± 17.5	116.2 ± 16.1	
BMI, kg/m^2^	40.3 ± 3.8	40.4 ± 3.4	
1 year after surgery			
Age, years	45.1 ± 10.1	45.6 ± 10.7	0.66
Bodyweight, kg	82.7 ± 15.5	78.2 ± 14.1	0.011
BMI, kg/m^2^	28.6 ± 4.2	27.2 ± 3.5	0.001
Total weight loss, kg	33.5 ± 10.6	38.0 ± 8.8	< 0.001
Total weight loss, %	28.9 ± 7.0	32.8 ± 6.5	< 0.001
BMI change, kg/m^2^	−11.6 ± 3.3	−13.2 ± 2.8	< 0.001

Results are given as mean ± SD or frequency (%)

### Vitamin and mineral supplementation

**Fig. 1:**
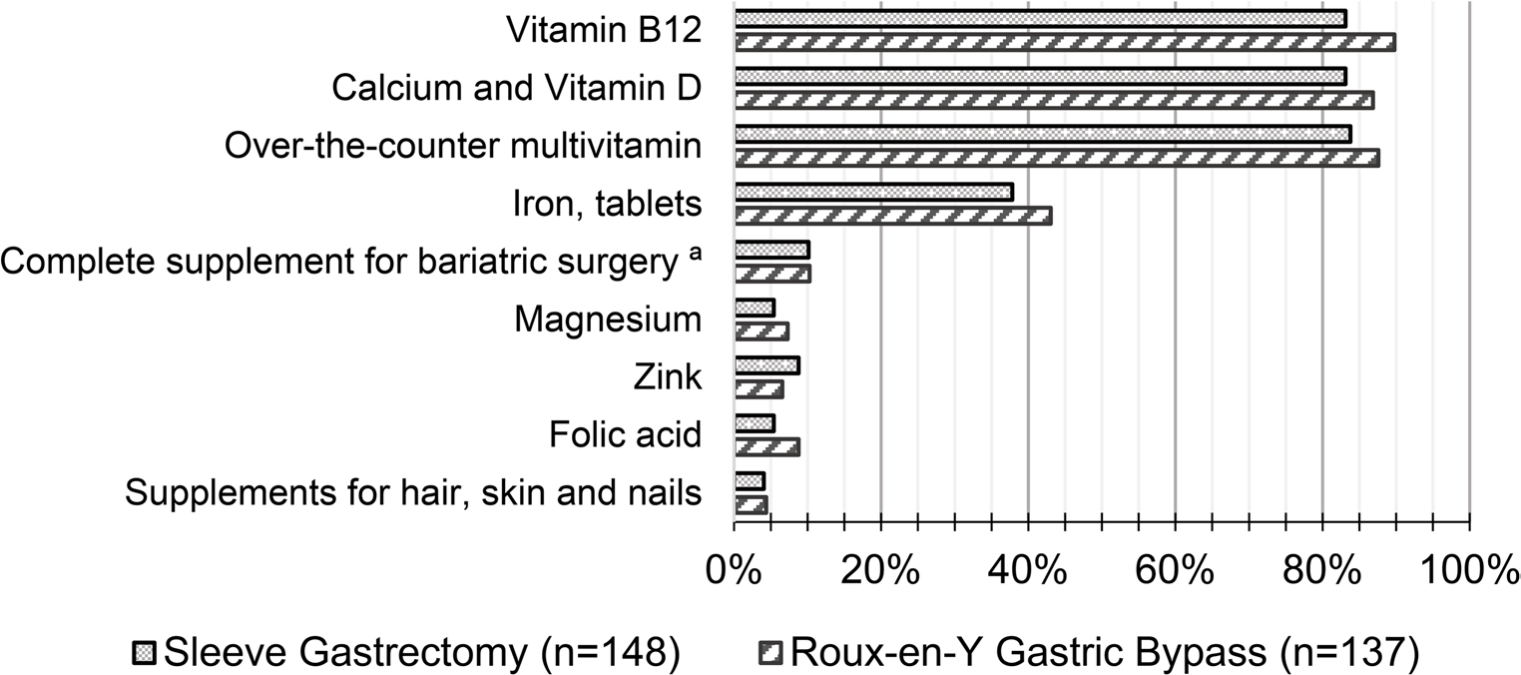
Proportion of participants who reported intake of supplements one year after surgery ^a^ A *specifically designed vitamin/mineral formula* for bariatric surgery

A total of 280 individuals, 98% (SG = 99%, RYGB = 98%) reported use of micronutrient supplements one year after surgery. There was one participant not using supplements, and missing data in four. Proportions of participants who reported intake of various supplements one year after surgery is shown in Fig. 1. The most used supplement was vitamin B12 (SG = 83%, RYGB = 90%) followed by calcium and vitamin D (SG = 83%, RYGB = 87%) and OTC multivitamin tablet (SG = 84%, RYGB = 88%). A specifically designed vitamin/mineral formula for bariatric surgery was used in 10% of participants after SG and RYGB.

### Dietary intake

Reported intake of energy and micronutrients from the diet one year after surgery in under-reporters and acceptable reporters is presented in Table 2 and in Supplementary Table 1**.** Acceptable reporters (40%, *n* = 114) were defined using the Goldberg cut-off, where the ratio between reported energy intake and BMR was between 0.92 and 2.1. No individuals were classified as over-reporters (reporting an intake above 2.1). Dietary under-reporters had a lower intake of micronutrients compared to those who reported an acceptable energy intake.

**Table 2:** Reported daily intake of energy and micronutrients from diet one year after surgery in under-reporters and acceptable reporters

Dietary intake assessed by 4-day food record	Overall (*n* = 285)	Under reporters (*n* = 171)	Acceptable reporters (*n* = 114)	*p*-values (under reporters vs. acceptable reporters)
Energy (kcal/day)	1330 ± 340	1100 ± 260	1660 ± 330	< 0.001
Vitamin A (RE)	523 ± 320	450 ± 320	627 ± 300	< 0.001
Vitamin B12 (µg)	3.7 ± 2	3.2 ± 1	4.4 ± 2	< 0.001
Vitamin C (mg)	55 ± 33	49 ± 30	64 ± 35	< 0.001
Vitamin D (µg)	6 ± 3	5 ± 3	7 ± 4	< 0.001
Calcium (mg)	632 ± 290	507 ± 190	820 ± 310	< 0.001
Folic acid (µg)	195 ± 69	167 ± 50	238 ± 70	< 0.001
Iron (mg)	6 ± 3	5 ± 2	7 ± 3	< 0.001

Results are given as mean ± SD

The reported dietary intake of micronutrients one year after surgery and in the comparison group, Swedish dietary habits [46], is presented in Table 3 and in Supplementary Tables 2–3. As expected, males had a higher body weight and energy intake than females, both in the comparison group and in patients undergoing both surgical procedures. This also applied to most micronutrients.

Table 3 and Supplementary Table 2 presents the reported intake from diet in comparison to Recommended Intake (RI) and Adequate Intake (AI) as specified in the Nordic Nutrition Recommendations 2023 for healthy adults aged 25–50 years [47]. RI is used when there is sufficient scientific evidence to determine the minimum intake needed to maintain nutritional status. AI is used when the evidence is insufficient and is usually based on the level of intake that does not lead to any adverse health effects in the population [47].

Compared to the comparison group, participants who had undergone SG or RYGB reported intake below the RI or AI for a greater number of micronutrients. Females who had undergone SG reported dietary intakes below the RI and AI for vitamin A, vitamin C, vitamin D, calcium, folate, iron, and magnesium. Compared to all other groups, females who had undergone SG had the highest number of micronutrient intake below the RI and AI.

**Table 3:** Reported intake of energy and micronutrients from diet one year after bariatric surgery. Under-reporters excluded

	Sleeve Gastrectomy (*n* = 50)		Roux-en-Y Gastric Bypass (*n* = 64)	
	Women (*n* = 42)	Men (*n* = 8)	Women (*n* = 52)	Men (*n* = 12)
Bodyweight (kg)	76 ± 4	96 ± 9	73 ± 11	83 ± 11
BMI (kg/m^2^)	27 ± 4	30 ± 5	26 ± 3	26 ± 3
Energy (kcal/day)	1600 ± 290	2070 ± 320	1600 ± 310	1900 ± 280
Vitamin A (RE)	576 ± 260^a^	676 ± 300^a^	656 ± 330^a^	647 ± 310^a^
Vitamin B12 (µg)	4 ± 2	5 ± 2	4 ± 2	5 ± 2
Vitamin C (mg)	52 ± 23^a^	60 ± 27^a^	71 ± 41^a^	72 ± 36^a^
Vitamin D (µg)	7 ± 4^a^	9 ± 5^a^	6 ± 3^a^	8 ± 5^a^
Calcium (mg)	798 ± 250^a^	1130 ± 310	772 ± 340^a^	898 ± 240^a^
Folate (µg)	213 ± 57^a^	307 ± 79^a^	232 ± 62	230 ± 78^a^
Iron (mg)	6 ± 2^a^	10 ± 7	6 ± 2^a^	9 ± 3

Results are given as mean ± SD

^a^ < Recommended daily Intake (RI)

### Intake of vitamin and minerals from supplementation and diet

The reported intake of micronutrients from diet and supplements, one-year after surgery, is presented in Table 4 and in Supplementary Table 5**.** A significant difference (*P* < 0.05) regarding the source of micronutrient intake is observed between SG and RYGB, except for vitamin A and calcium, where about half of the intake came from diet and supplements, respectively, both after RYGB and SG. After SG and RYGB, most participants reported supplement intake consistent with the recommendations.

The RYGB group reported a higher intake of vitamin A and folic acid from supplementation, as well as a higher intake of vitamin C from diet. Additionally, the RYGB group showed a greater total intake (from both diet and supplementation) of folic acid. There were no differences in energy intake between the groups, nor were there any significant differences in the intake of other vitamins and minerals.

**Table 4:** Reported intake of energy and micronutrients from diet, supplements and total intake one year after surgery. Under-reporters excluded

	Sleeve Gastrectomy (*n* = 50)		Roux-en-Y Gastric Bypass (*n* = 64)		Difference between Sleeve Gastrectomy and Roux-en-Y Gastric Bypass (*p*-value)		
Energy (kcal/day)	1676 (± 260)		1651 (± 330)		0.721		
	Diet	Supplements	Diet	Supplements	Diet	Supplements	Diet + Supplements
Vitamin A (RE)	592 ± 260	580 ± 200	655 ± 320	656 ± 100	0.27	**0.017**	0.38
Vitamin B12 (µg)	4 ± 2	874 ± 280	4 ± 2	942 ± 250	0.906	0.19	0.16
Vitamin C (mg)	53 ± 23	87 ± 33	72 ± 40	92 ± 23	**0.001**	0.33	0.07
Vitamin D (µg)	7 ± 4	802 ± 460	6 ± 3	803 ± 430	0.42	1.00	1.00
Calcium (mg)	851 ± 280	931 ± 260	796 ± 330	887 ± 270	0.34	0.40	0.27
Folic acid (µg)	228 ± 69	374 ± 140	245 ± 70	436 ± 130	0.22	**0.014**	**0.005**
Iron (mg)	7 ± 4	50 ± 54	7 ± 2	52 ± 51	0.88	0.83	0.76

Results are given as mean ± SD

## Discussion

In this exploratory cross-sectional multicenter sub-study of a randomized trial comparing SG and RYGB, the intake of vitamins and minerals from diet and supplements was, in general, similar between patients operated with SG and RYGB at one year after surgery and not significantly different from a non-operated normal weight comparison group.

When assessed separately in surgical patients, the majority of micronutrient intake came from supplements, except for vitamin A and calcium, where about half of the intake came from diet and supplements, respectively without differences between SG and RYGB. The calcium intake from diet in the current study is in line with the results from the randomized single-site study by Barstad, et al. [48] and the study by Moizé et al. [49]. The reported intake of vitamin A from the diet was higher in the study by Barstad, et al. [48] than in this study and in the Swedish normal weight comparison group [46]. This might be attributed to differences in food culture, dietary habits, product enrichment, or methodology.

Traditionally, post-bariatric surgery nutritional guidelines focus mainly on the intake of vitamins and minerals through supplementation rather than dietary recommendations [15, 50]. Consistent with previous research [25, 49], our findings show that intake of several vitamins and minerals from diet remain below recommended levels, which is concerning given the reduced intestinal absorption after surgery. These results suggest that dietary management may warrant a more prominent role, potentially having a more significant impact than previously assumed. A focus on total dietary intake, including both supplements and diet would likely help to optimize and individualize treatment in clinical practice.

The reported energy intake was similar between the surgical methods. A lack of difference in energy intake between SG and RYGB, despite a higher weight loss after RYGB, may indicate other mechanisms of action e.g. difference in postprandial energy expenditure, in addition to reduction in energy intake and/or uptake [51–53].

The somewhat lower intake of vitamin C after SG compared to RYGB without significant differences in the intake of calcium, magnesium, and phosphorus from the diet is in line with previous studies [48, 49]. The difference in vitamin C intake from diet corresponds to approximately one-third of an orange or 1 dl of raspberries [36].

The current study indicates a lower intake of folic acid and vitamin A from supplements after SG compared to RYGB, as well as a lower total intake of folic acid. Although statistically significant, the differences in intake are small. The study by Barstad, et al. did not calculate supplement intake separately, but the overall lower intake of folic acid after SG is in corroboration with their results [25]. Since participants in both groups were prescribed the same supplementation regimen, it is surprising to observe a difference in reported intake of vitamin A and folic acid from supplements between the surgical methods. Future studies should assess potential differences in attitudes and preconceptions about the need for supplementation between the surgical methods.

Overall, participants reported an excellent use of micronutrient supplement. The supplements with the most common use were vitamin B12, calcium, vitamin D, and multivitamins, which are recommended in the Nordic guidelines [14]. A high rate of adherence to supplements one year after surgery are consistent with results in previous studies [54, 55] and a high level of initiation rate after surgery indicates that there are few early barriers to supplement use [56].

Strengths of this study include randomized allocation to SG and RYGB, which eliminates systematic bias at baseline, the multicenter setting, as well as the use of dual methods for dietary intake (food diaries followed by a check-up by a dietitian). Furthermore, the population-based sampling and high coverage offer a representative sample, presumably being generalizable to a broader bariatric population.

However, this study has several limitations including self-reported dietary intake, use of supplements and that pre-operative assessments are lacking. Measuring dietary intake is challenging, and all dietary assessment methods have advantages and disadvantages. Underreporting of dietary intake is common [57], and the methodological limitations related to the 4-day food diary include its time-consuming nature. In this study, some participants declined participation for this reason. Additionally, the awareness of recording food intake may, consciously or subconsciously, influence eating behaviors and certain foods or meals may be omitted. Eating habits, dietary norms, and food choices as well as use of food enrichments can vary between different countries, ethnicities, and cultures [58] which might further limit the generalizability of the results. Furthermore, as this study was conducted only one year after the surgery, the findings may not be applicable in the longer-term.

## Conclusions

The reported energy intake after SG and RYGB one year after surgery was similar, despite differences in the reduction of body weight. Reported intake of vitamins and minerals from diet and supplements was, in general, similar and satisfactory one year after SG and RYGB. However, after SG, patients reported somewhat lower dietary intake of vitamin C, vitamin A and folic acid from supplements compared to after RYGB. Longer follow-up should address whether these differences will increase over time.

## Supplementary Information

Below is the link to the electronic supplementary material.

Supplementary Material 1 (210 KB)

## Data Availability

The data supporting the findings of this study are not publicly available due to ongoing analyses within a related research project.

## Abbreviations

AI: Adequate intake
BEST: Bypass Equipoise Sleeve Trial
BMR: Basal Metabolic Rate
Kcal: Kilocalories
MBS: Metabolic and Bariatric Surgery
mg: Milligram
OTC: Over-the-counter
PAL: Physical Activity Level
RE: Retinol Equivalents
RI: Recommended intake
RYGB: Roux-en-Y Gastric Bypass
SG: Sleeve Gastrectomy
SOReg: Scandinavian Obesity Surgery Registry
µg: Microgram
