# Supplementary material for: Energy and Micronutrient Intake One Year After Sleeve Gastrectomy Versus Roux-en-Y Gastric Bypass: A Substudy of the Bypass Equipoise Sleeve Trial
Source: Obes Surg. 2026 Mar 6;36(4):1628–36. doi: 10.1007/s11695-026-08550-3 (PMC13083348; doi:10.1007/s11695-026-08550-3)
Supplement: Supplementary file 1 — Supplementary Material 1 (210 KB) [file 11695_2026_8550_MOESM1_ESM.pdf]

Supplementary material

ENERGY AND MICRONUTRIENT INTAKE ONE YEAR AFTER SLEEVE GASTRECTOMY VERSUS ROUX-EN-Y GASTRIC BYPASS: A Substudy of the Bypass Equipoise Sleeve Trial

Supplementary table 1. Reported daily intake of energy and micronutrients from diet one year after surgery in under-reporters and acceptable reporters

Dietary intake assessed by 4-day food record	Overall (n=285)	Under reporters (n=171)	Acceptable reporters (n=114)	p-values (under reporters vs. acceptable reporters
Vitamin B1, Thiamnine (mg)	0.9±0.4	0.8±0.3	1.1±0.5	<0.001
Vitamin B2, Riboflavin (mg)	1.1±0.5	0.9±0.3	1.4±0.5	<0.001
Vitamin B3, Niacin (mg)	14±7	12±5	17±8	<0.001
Vitamin B6, Pyridoxine (mg)	1.3±0.9	1.1±0.5	1.7±1.3	<0.001
Vitamin E (µg)	9±4	8±3	11±4	<0.001
Vitamin K (µg)	50±34	44±29	59±38	<0.001
Potassium (mg)	1820±600	1560±410	2200±630	<0.001
Selenium (µg)	35±3	32±13	41±18	<0.001
Sodium (mg)	2100±670	1830±540	2500±650	<0.001
Zinc (mg)	7±3	7±2	10±3	<0.001
Magnesium (mg)	193±71	162±44	239±78	<0.001
Phosphorus (mg)	1040±350	872±225	1300±35	<0.001

Results are given as mean ± SD

Supplementary table 2. Reported intake of energy and micronutrients from diet one year after bariatric surgery (under-reporters excluded)

	Sleeve Gastrectomy (n= 50)		Roux-en-Y Gastric Bypass (n=64)	
	Women (n=42)	Men (n=8)	Women (n=52)	Men (n=12)
Vitamin B1(mg)	1±0.4	1±0.7	1±0.4	2±0.6
Vitamin B2(mg)	1±0.5	2±0.6	1±0.5	2±0.5
Vitamin B3(mg)	16±8	20±13	16±7	20±9
Vitamin B6(mg)	2±1	2±1	2±1	2±1
Vitamin E (mg)	11±4	16±5	11±4	12±3
Vitamin K (µg)	48±20	78±40	63±50	70±40
Potassium (mg)	2020±650	2720±600	2170±540	2650±580
Selenium (µg)	40±19	55±25	38±16	48±16
Sodium (mg)	2360±540	3160±560	2420±680	2900±580
Zinc (mg)	9±2	13±4	9±3	13±4
Magnesium (mg)	219±54[a]	332±150[a]	227±67[a]	296±64[a]
Phosphorus (mg)	1240±280	1790±360	1220±330	1500±290

Results are given as mean ± SD [a] < Adequate daily intake (AI)

Supplementary table 3. Reported intake of energy and micronutrients from diet in Swedish dietary habits.

	Swedish dietary habits, comparison group (n=1430)	
	Women (n=807)	Men (n=623)
Bodyweight (kg)	68±3	82±3
BMI (kg/m^2)	25±4	26±4
Energy (kcal/day)	1800±520	2000±690
Vitamin A (RE)	736±860	770±460[a]
Vitamin B12 (µg)	5±3	6±3
Vitamin C (mg)	91±56[a]	86±51[a]
Vitamin D (µg)	8±5[a]	7±5[a]
Calcium (mg)	820±330[a]	968±410
Folate (µg)	244±94[a]	259±92[a]
Iron (mg)	10±3[a]	12±4
Magnesium (mg)	303±96	364±120
Phosphorus (mg)	1670±370	1670±480

Results are given as mean ± SD [a] < Recommended daily Intake (RI)

Supplementary table 4. Recommended intake of vitamin and minerals from diet and supplements.

	Recommended Intake (RI) for healthy adults [1]	Daily recommendation of micronutrient supplementation after metabolic and bariatric surgery [2]
Vitamin A (RE)	700 (women), 800 (men)	OTC [a] multivitamin/mineral tablet contains vitamin A
Vitamin B12 (µg)	4	350-1000
Vitamin C (mg)	95 (women), 110 (men)	No official recommendation available
Vitamin D (µg)	10	At least 20, in combination with calcium
Calcium (mg)	950	At least 500, in combination with vitamin D
Folic acid (µg)	330	OTC [a] multivitamin/mineral tablet contain 400. An additional 500 is recommended during the first 12 weeks of pregnancy
Iron (mg)	15 (women), 9 (men)	45-60 and OTC [a] multivitamin/mineral tablet contains iron
	Adequate Intake (AI) for healthy adults[1]	
Magnesium (mg)	300 (women), 350 (men)	OTC [a] multivitamin/mineral tablet contains magnesium
Phosphorus (mg)	520	No official recommendation available

[a] Over-the-counter (OTC)

Supplementary table 5. Reported intake of energy and micronutrients from diet, supplements and total intake one year after surgery. Under-reporters excluded.

	Sleeve Gastrectomy (n= 50)		Roux-en-Y Gastric Bypass (n=64)		Difference between Sleeve Gastrectomy and Roux-en-Y Gastric Bypass (p-value)		
	Diet	Supple-ments	Diet	Supple-ments	Diet	Supple-ments	Diet + Supplements
Vitamin B1 (mg)	1±0	1±1	1±0	1±0	1.0	0.26	0.50
Vitamin B2 (mg)	1±1	1±0	1±0	1±0	0.63	0.11	0.58
Vitamin B3 (mg)	16±9	15±5	17±7	16±2	0.68	0.23	0.32
Vitamin B6 (mg)	2±2	1±0	2±1	1±0	0.72	0.12	0.92
Vitamin E (µg)	12±4	12±6	11±4	12±5	0.14	0.70	0.20
Vitamin K (µg)	53±26	39±38	65±45	56±33	0.11	0.003	0.001
Potassium (mg)	2130±690	missing	2260±580	missing	0.29	-	-
Selenium (µg)	43±20	55±21	40±16	59±15	0.40	0.33	0.99
Sodium (mg)	2480±610	missing	2520±680	missing	0.78	-	-
Zinc (mg)	10±3	12±13	9±3	11±11	0.92	0.69	0.97
Magnesium (mg)	237±85	109±68	240±71	92±47	0.81	0.11	0.43
Phosphorus (mg)	1327±350	missing	1276±340	missing	0.44	missing	missing

Results are given as mean ± SD

References

1. Blomhoff.R.et al. *Nordic Nutrition Recommendations 2023*. 2023: Copenhagen: Nordic Council of Ministers.
2. Laurenius, A., et al., *Nordiska riktlinjer för kosttillskott och uppföljning efter obesitaskirurgi - Monitorering och supplementering med vitaminer och mineraler* Lakartidningen, 2018. **115**.
